# Endogenous endophthalmitis caused by *Streptococcus mitis*: A case report

**DOI:** 10.1097/MD.0000000000039096

**Published:** 2024-07-26

**Authors:** Saki Nakayama, Hideya Itagaki, Yoshinobu Abe, Nobutoshi Matumura, Tomoyuki Endo

**Affiliations:** a School of Medicine, Tohoku Medical and Pharmaceutical University, Sendai, Miyagi, Japan; b Division of Emergency and Disaster Medicine, Tohoku Medical and Pharmaceutical University Hospital, Sendai, Miyagi, Japan.

**Keywords:** cataracts, endocarditis, endogenous endophthalmitis, *Streptococcus mitis*

## Abstract

**Rationale:**

Endogenous endophthalmitis is a rare disease caused by hematogenous intraocular metastasis of bacteria from an infectious source. Diagnosing endogenous endophthalmitis is challenging for non-ophthalmologists. However, ophthalmic diseases can cause irreversible vision loss, making prompt diagnosis and treatment critical. Here we present a rare case of endogenous endophthalmitis initially misdiagnosed as a cataract.

**Patient concerns:**

An 84-year-old Japanese man presented to the emergency department with fever and dysmotility. The patient was aware of a left subconjunctival hemorrhage and cloudy cornea upon arrival at the hospital, but he misunderstood it as a fall-induced subconjunctival hemorrhage and age-related cataracts.

**Diagnoses:**

On the day following admission, petechial hemorrhage on the eyelid conjunctiva and the detection of *Streptococcus mitis* in the blood culture results led us to suspect endophthalmitis rather than cataracts. A definitive diagnosis of endophthalmitis was made through ophthalmologic examinations, and endophthalmitis was considered secondary to endocarditis.

**Interventions:**

Subsequently, antimicrobial treatment was continued.

**Outcomes:**

However, the patient developed myocardial infarction and died on the ninth day of hospitalization.

**Lessons:**

Two important lessons were learned from the examination of this case of endogenous endophthalmitis caused by *S mitis*. First, endophthalmitis and cataracts can be misdiagnosed. Because the symptoms of endophthalmitis and cataracts, such as decreased vision, photophobia, and blurred vision, are similar, the eye must be cautiously examined. Second, endocarditis caused by *S mitis* may lead to endogenous endophthalmitis. Although *S mitis* is not pathogenic, endogenous endophthalmitis may occur in patients with certain risk factors, such as older age, cancer, and immunosuppression.

## 1. Introduction

Endophthalmitis is an intraocular infection of the ocular capsule with diffuse vitritis.^[1]^ It is an emergent ophthalmic disease that can cause irreversible vision loss and is classified as either exogenous or endogenous.^[1]^ Exogenous endophthalmitis is caused by direct intraocular invasion of bacteria, such as during surgery or trauma. In contrast, endogenous endophthalmitis, also known as metastatic endophthalmitis, is caused by the hematogenous transfer of bacteria into the eye from a remote organ (the primary source of infection) and is a relatively rare disease.^[2–4]^ Endogenous endophthalmitis is generally associated with potential immunosuppression as a risk factor for diabetes mellitus, autoimmune diseases, drug toxicity, and malignant tumors.^[3,5,6]^ The most common cause of endogenous endophthalmitis is liver abscess, followed by pneumonia, meningitis, and endocarditis.^[3,5,6]^ The most common causative organisms are *Staphylococcus aureus* and *Streptococcus beta* among Gram-positive cocci and *Klebsiella pneumoniae* among Gram-negative rods.^[1,3,5,7]^ Although *Streptococcus mitis* is a major causative agent of endocarditis, it rarely causes endogenous endophthalmitis, and only a few cases have been reported in the literature.^[3,5,7,8]^

In this report, we describe a case of endophthalmitis caused by *S mitis* that was initially mistaken for *a cataract* during the initial examination but later diagnosed as endogenous endophthalmitis.

## 2. Case presentation

The Tohoku Medical and Pharmaceutical University Ethics Committee approved this study (approval number: 2023-4-047). The patient and his family provided written informed consent to publish this case report and accompanying images.

**Figure 1.**
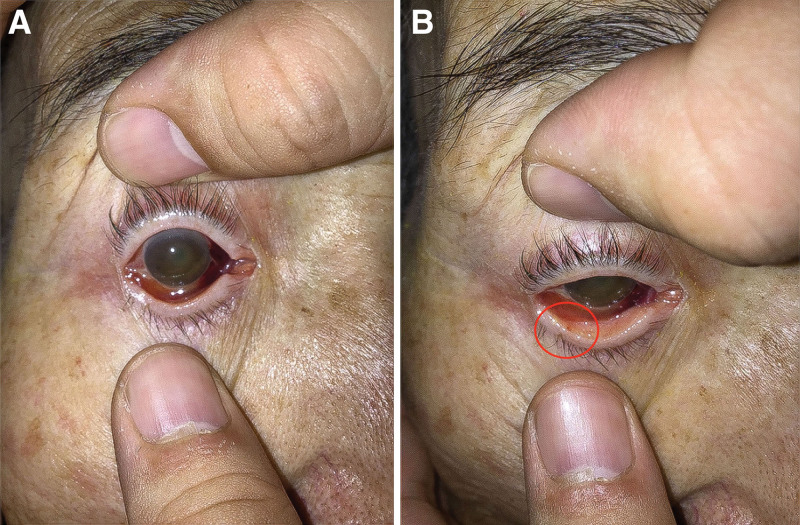
(A) Subconjunctival hemorrhage and cloudy cornea. (B). Petechial hemorrhages on the eyelid conjunctiva (red circle).

An 84-year-old man with fever and dyspnea was admitted to our hospital via emergency medical services. He had been ataxic for 6 days before presenting to the hospital and had experienced multiple falls at home. Two days before visiting our hospital, the patient had consulted his local doctor, who diagnosed him with a common cold. Upon arrival, his family found him unable to get up after defecating in the toilet and brought him to our hospital for emergency care. The patient had a medical history of hypertension, chronic heart failure, an ascending aortic aneurysm, and cataracts and was currently on telmisartan, amlodipine besilate, aspirin, vonoprazan fumarate, ezetimibe, and atorvastatin calcium hydrate. His vital signs at admission were as follows: blood pressure, 109/52 mm Hg; pulse, 71 beats/min; body temperature, 39.3°C; respiratory rate, 20 breaths/min; and oxygen saturation, 95%. The left subconjunctival hemorrhage was due to trauma from a fall, and the cloudy cornea was associated with cataracts (Fig. 1A).

Other findings included systolic ejection murmur on chest auscultation. Blood tests revealed an elevated inflammatory response (white blood cell count, 20,300 cells/μL; C-reactive protein, 15.58 mg/dL; and procalcitonin, 71.7 ng/mL), anemia (hemoglobin, 6.5 g/dL), and decreased renal function (blood urea nitrogen, 96 mg/dL and creatinine, 3.41 mg/dL). Point-of-care ultrasound showed good echocardiographic contraction, no wall motion abnormality, and increased brightness of the aortic valve; however, verrucae were not observed, which suggested endocarditis. Contrast-enhanced computed tomography was also performed, but no obvious source of infection was noted. Blood cultures were performed because of the high degree of inflammatory reactions. Based on these results, the patient was diagnosed with sepsis and acute renal failure. However, the source of the infection was unknown, and the patient was admitted to the hospital on the same day. The following day, another staff member interviewed the patient and learned that he had previously undergone cataract surgery and had been experiencing visual difficulty for several days before hospital admission. Physical examination revealed petechial hemorrhages in the clouded cornea and eyelid conjunctiva (Fig. 1B). On the basis of these findings, we suspected that the left subconjunctival hemorrhage and cloudy cornea were not due to cataracts or trauma but rather to endophthalmitis caused by endocarditis. Subsequent blood culture results revealed 2/2 sets of *S mitis*. Echocardiography was repeated on the same day, and verrucae were identified on the aortic and mitral valves, leading to the diagnosis of infective endocarditis. Ophthalmological examination revealed endophthalmitis, and the patient was diagnosed with endocarditis-induced endogenous endophthalmitis. On the fifth day after admission, antibiotics were modified from tazobactam and piperacillin to penicillin G, which improved the inflammatory response. However, on the seventh day of admission, the patient experienced chest symptoms, and an electrocardiogram revealed ST-segment elevation in I and aVL, ST-segment depression in V2–4, and decreased wall motion in the lateral and posterior walls, suggesting ST-elevation myocardial infarction in the left circumflex branch. The cardiology and cardiovascular surgery departments at our hospital indicated that surgery would be challenging. Consequently, the patient was treated palliatively and died on the ninth day of the illness.

## 3. Discussion

We encountered a case in which the initial cataract diagnosis was attributed to endogenous endophthalmitis caused by *S mitis*-induced endocarditis and learned that endophthalmitis and cataracts can be misdiagnosed, and that *S mitis*-induced endocarditis can cause endogenous endophthalmitis.

The incidence of endogenous endophthalmitis is 0.04% to 0.4%, accounting for 2% to 15% of all cases of endophthalmitis.^[3,4]^ Endogenous endophthalmitis arises from the hematogenous transport of the causative organism from a remote organ, the infection site, and its passage through the blood-ocular barrier.^[3,5]^ Mortality rates increase by up to 29%, suggesting that ocular involvement is a good predictor of mortality in patients with systemic disease.^[9]^ Visual prognosis is poor, with only 32% of patients maintaining better visual acuity than the index valve, 44% losing the photoreceptor valve, and 25% requiring ocular resection.^[10]^ Regarding the age of onset, previous studies have reported that the disease is most common at approximately 50 years of age.^[10]^ Another study reported a relatively young age range, with a mean age of 35 years, and 24 of the 72 reported cases were younger than 20 years.^[11]^ Based on these 2 reports, endogenous endophthalmitis is thought to occur at a relatively young age. More than 60% of patients with endogenous endophthalmitis are at risk of some degree of immunosuppression, predisposing them to infection.^[3,5,6,12]^ Diabetes mellitus was found to be the most common risk factor, followed by malignancy, autoimmune diseases, immunosuppressive drug therapy, and intravenous drug use.^[3,5,6,12]^ The most common comorbidities reported in patients with endophthalmitis are gastrointestinal disorders, hypertension, cardiac disease, and cerebral infarction.^[2]^ Typical symptoms include blurred vision (89%), pain (49%), redness, and photophobia, and clinical ocular signs such as loss of fundus imaging (40%), decreased pupil size (35%), vitreous (33%), and anterior chamber inflammation (32%).^[5]^ Most patients with endogenous endophthalmitis have these risk factors, although some reports have stated that approximately 5% of patients present without underlying diseases.^[13]^

Our patient was 84 years old, which is an atypical age for endogenous endophthalmitis. He had no systemic risk factors for endogenous endophthalmitis, such as diabetes mellitus or immunosuppressive diseases. Previous studies have suggested that the interaction between innate and acquired immunity is associated with the development of hypertension.^[14]^ Hence, the possibility that age and history of hypertension affected the patient immunity cannot be ruled out in this case.

We diagnosed the patient with cataracts on the basis of the presence of a cloudy cornea on admission. Cataracts occur when the vitreous body inside the eye becomes cloudy, resulting in vision loss. Photophobia, blurred vision, and vision loss may occur as the disease progresses. The symptoms were similar to those of endophthalmitis; however, because cataract entails lens clouding, no blurring of the pupil outline was observed, as in our case. In this study, the contour of the pupil was blurred because of anterior chamber inflammation, possibly induced by conjunctival hyperemia caused by inflammation. Endophthalmitis is challenging to diagnose because of its infrequent and nonspecific clinical manifestations. Some authors have reported that the diagnosis is incorrect in 22% to 33% of patients with endogenous endophthalmitis, and the percentage is probably even higher if unreported cases are included.^[5,10,15]^ In our case, another staff member recognized the misdiagnosis by conducting a detailed interview and diagnosed the patient with endogenous endophthalmitis.

After the diagnosis of endogenous endophthalmitis, identifying its cause is essential. In addition to liver abscesses, urinary tract infections, pneumonia, and skin and soft tissue infections, endocarditis is a common cause.^[3]^ Indeed, Jenkins et al reported that 37% (13/35) of patients with endogenous endophthalmitis had endocarditis.^[2,5]^ Moreover, Jackson et al reported that the most common cause of endogenous endophthalmitis was liver abscess (67/392; 19%), with other Asian studies from Korea and Singapore also showing a similar trend.^[12,13,16]^ Gram-negative bacteria are the most frequent cause of endogenous endophthalmitis in East Asia, accounting for 22.2% to 77.1% of all endogenous endophthalmitis cases, while Klebsiella is the most common organism, accounting for 31.7% to 87.6% of all cases in Southeast Asia.^[17]^ *S mitis*, detected in the blood cultures of our patient, is a type of alpha-hemolytic streptococcus belonging to the Viridans group of streptococci, which is endemic to the oral flora. The Viridans group of streptococci is a common bacterium that causes infections and is the second most common organism causing endocarditis.^[7]^ Okada et al reported that the frequency of endogenous endophthalmitis caused by streptococci was 10.7% (3/28 patients), whereas Jacson et al reported a frequency of 2.6% (9/342 patients).^[2,5]^ Among these cases, only 12 were caused by *S mitis*, and only 2 were caused by endocarditis, including our case (Table 1).^[4,9,18–24]^ Although *S mitis* has a low virulence, it can cause infections such as bacteremia and endocarditis in patients with risk factors such as older age, cancer, and immunosuppression.^[25]^ Of the 12 cases of endogenous endophthalmitis caused by *S mitis*, 11 had immunosuppressive factors, such as advanced age, diabetes mellitus, or leukemia, with the exception of a 3-year-old patient. Understanding that *S mitis* causes endophthalmitis is essential and should be monitored in patients with immunosuppressive factors and those with risk factors that may affect immunity, such as aging and hypertension.

**Table 1:** Clinical characteristics and treatment outcomes of 13 cases of endogenous endophthalmitis due to *Streptococcus mitis*.

Author	Sex	Age (y)	Immunosuppressive risk factor	Medical condition	Sources of infection	Laterality and visual acuity	Eye symptoms	Culture (B/V)	Vitrectomy	Intravitreal antibiotics	Systemic antibiotics	Visual outcome	Systemic outcome
Mavrakanas et al.^[18]^	F	68	Type 2 diabetes mellitus	Coronary artery disease	Food osteomyelitis	OS	Blurred vision redness pain	Negative/S. mitis	No	Yes	Yes	Loss (evisceration of the eye)	Discharge
Liu et al.^[19]^	F	67	None	Hypertension	Community-acquired pneumonia	OS	Visual loss pain	S. mitis/S. mitis	Yes	Yes	Yes	Loss (evisceration of the eye)	Discharge
Harrison et al.^[20]^	F	3	None	None		OS	Redness irritated eye	S. mitis/S. mitis	No	Yes	Yes	Recovery	Discharge
Dinani et al.^[21]^	M	85	Diabetes mellitus	Hypertension	Endocarditis	OD	Pain redness visual loss	Alpha-hemolytic streptococcus/negative	No	Yes	Yes	Recovery	Discharge
Ono et al.^[22]^	M	33	Acute myeloid leukemia	Tonsillectomy and recurrent sinusitis	Unknown	OS + OD	Pain visual loss	Alpha-hemolytic streptococcus/negative	Yes	Yes	Yes	Recovery	Discharge
Reed and Hibberd^[23]^	M	71	None	None	Endoscopy	OS	Blurred vision pain visual loss	Negative/S. mitis	No	No	Yes	Recovery	Discharge
Schiedler et al.^[9]^	M	42	Diabetes mellitus	Hemodialysis kidney/pancreas transplant	Kidney abscess	OD	Unknown	Negative/Streptococcus oralis	Unknown	Unknown	Unknown	20/800 (0.025)	Unknown
Chen et al.^[24]^	F	80	None	Age-related macular edema	Vitreous	OD	Visual loss redness	Unknown/S. mitis	Yes	No	Unknown	Hand motions	Unknown
Our case	M	84	None	Hypertension ascending aortic aneurysm chronic heart failure	Endocarditis	OD	Blurred vision redness	S. mitis/NP	No	Yes	Unknown	Counting fingers	Unknown

B = Blood culture, F = Female, M = Male, NP = Not performed, OD = Right eye, OS = Left eye, V = Vitreous culture.

Cataracts are highly prevalent among older adults, and their ocular findings are similar to those of endogenous endophthalmitis. Therefore, in cases of suspected or confirmed bacteremia accompanied by ocular symptoms, a detailed ocular examination and urgent examination by an ophthalmologist are crucial. The causative agent in this case, *S mitis*, can cause endogenous endophthalmitis, although this is rare; therefore, caution should be exercised when patients complain of decreased or blurred vision.

## Author contributions

**Conceptualization:** Hideya Itagaki.

**Data curation:** Hideya Itagaki, Yoshinobu Abe.

**Formal analysis:** Hideya Itagaki.

**Funding acquisition:** Hideya Itagaki.

**Investigation:** Hideya Itagaki.

**Methodology:** Hideya Itagaki.

**Project administration:** Hideya Itagaki.

**Resources:** Hideya Itagaki.

**Software:** Hideya Itagaki.

**Supervision:** Hideya Itagaki.

**Validation:** Hideya Itagaki.

**Visualization:** Hideya Itagaki.

**Writing – original draft:** Saki Nakayama, Hideya Itagaki.

**Writing – review & editing:** Hideya Itagaki, Nobutoshi Matumura, Tomoyuki Endo.
